# Kv4.2 potassium channels segregate to extrasynaptic domains and influence intrasynaptic NMDA receptor NR2B subunit expression

**DOI:** 10.1007/s00429-012-0450-1

**Published:** 2012-08-30

**Authors:** Walter A. Kaufmann, Ko Matsui, Andreas Jeromin, Jeanne M. Nerbonne, Francesco Ferraguti

**Affiliations:** 1 Department of Pharmacology, Innsbruck Medical University, Peter-Mayr Strasse 1a, 6020 Innsbruck, Austria; 2 Division of Cerebral Structure, National Institute for Physiological Sciences, 444-8787, Myodaiji, Okazaki, Japan; 3 NextGen Sciences DX, Boston, MA 02110, USA; 4 Department of Developmental Biology, Washington University Medical School, St. Louis, MO, 63110, USA

**Keywords:** Voltage-gated potassium channel, Immuno-electron microscopy, Freeze-fracture replica, Nearest neighbor analysis, Glutamatergic synapse

## Abstract

Neurons of the intercalated cell clusters (ITCs) represent an important relay site for information flow within amygdala nuclei. These neurons receive mainly glutamatergic inputs from the basolateral amygdala at their dendritic domains and provide feed-forward inhibition to the central nucleus. Voltage-gated potassium channels type-4.2 (Kv4.2) are main players in dendritic signal processing and integration providing a key component of the A currents. In this study, the subcellular localization and distribution of the Kv4.2 was studied in ITC neurons by means of light- and electron microscopy, and compared to other types of central principal neurons. Several ultrastructural immunolocalization techniques were applied including pre-embedding techniques and, most importantly, SDS-digested freeze-fracture replica labeling. We found Kv4.2 densely expressed in somato-dendritic domains of ITC neurons where they show a differential distribution pattern as revealed by nearest neighbor analysis. Comparing ITC neurons with hippocampal pyramidal and cerebellar granule cells, a cell type- and domain-dependent organization in Kv4.2 distribution was observed. Kv4.2 subunits were localized to extrasynaptic sites where they were found to influence intrasynaptic NMDA receptor subunit expression. In samples of Kv4.2 knockout mice, the frequency of NR1-positive synapses containing the NR2B subunit was significantly increased. This indicates a strong, yet indirect effect of Kv4.2 on the synaptic content of NMDA receptor subtypes, and a likely role in synaptic plasticity at ITC neurons.

## Introduction

The intercalated cell clusters (ITCs) of the amygdala are composed of densely packed GABAergic neurons that are distributed around the basolateral complex (Busti et al. 2011; Millhouse 1986). ITC neurons are characterized by a small soma (8–19 μm in diameter), a flattened dendritic tree and high density of dendritic spines (Pape and Pare 2010). These neurons strongly express μ-opioid and dopamine type I receptors (Jacobsen et al. 2006; Likhtik et al. 2008), and they are uniform with respect to active and passive membrane properties (Busti et al. 2011). They display a very high input resistance (~600 MΩ) and can sustain high firing rates with only modest spike frequency accommodation (Geracitano et al. 2007).

ITC neurons represent a crucial relay site for information flow within amygdala nuclei, receiving mostly glutamatergic inputs from the basolateral complex and providing feed-forward inhibition to the central nucleus (Royer et al. 1999). As in other types of neuron, incoming information is largely conveyed through synapses onto dendritic domains, where integration and processing of this information are crucially governed by the presence of certain types of K^+^ channels. Voltage-gated K^+^ (Kv) channels of the *shal*-type (Kv4) are main players in dendritic signal processing known to regulate excitability, signal integration and long-term potentiation (LTP; Birnbaum et al. 2004; Chen et al. 2006; Jan and Jan 1997). Kv4 channels are key components of K^+^ channels underlying the A currents (*I*_*A*_). They are fast inactivating channels that activate and inactivate at subthreshold potentials and recover from inactivation at a faster rate than other Kv channels (Serôdio et al. 1996). They prevent initiation of dendritic action potentials, limit back-propagation of action potentials into dendrites and modulate excitatory postsynaptic potentials (Cai et al. 2004; Hoffman et al. 1997; Johnston et al. 2003). Since they operate in a voltage range where other channels are closed, even subtle changes in the activity of these channels can have large repercussions in the overall excitability and firing properties of the cells expressing them.

Three distinct genes have been identified that encode the mammalian *shal*-homologs, namely Kv4.1, Kv4.2 and Kv4.3. In addition to the pore-forming subunits, native *shal*-channels require auxiliary ß-subunits to function, including potassium channel interacting proteins (KChIPs) and dipeptidyl-peptidase-like proteins (DPPs; An et al. 2000; Cui et al. 2008). In the mammalian brain, only Kv4.2 and Kv4.3 isoforms are abundant and they are differentially distributed both regionally and compartmentally (Covarrubias et al. 2008; Menegola et al. 2008; Serôdio and Rudy 1998). The amygdala was shown to possess high levels of Kv4.3, but small to negligible Kv4.2 mRNA expression (Serôdio and Rudy 1998). However, a recent immunocytochemical study reported an exceptionally high density of Kv4.2 in the ITCs when analyzing the distribution of Kv4 subunits in the amygdala basolateral complex (Dabrowska and Rainnie 2010). Since ITCs were immunonegative for Kv4.1 and Kv4.3 subunits, homomeric Kv4.2 channels likely prevail in these areas.

Data on the subcellular distribution of Kv4 isoforms are divergent, especially when it comes to the ultrastructural localization of Kv4.2. While some groups reported clustering of Kv4.2 subunits at synaptic specializations in central neurons (Alonso and Widmer 1997; Jinno et al. 2005), others showed Kv4.2 clustering at extrasynaptic sites (Kollo et al. 2006, 2008) or a non-homogeneous distribution along the proximo-distal axis of neuronal dendrites without the formation of any cluster (Kerti et al. 2011).

Amygdala neurons are known as sites of associative plasticity for Pavlovian fear memories, and formation of such memories includes activity-dependent changes in the strength of synapses (Pape and Pare 2010). While fear conditioning likely results from potentiation of synaptic inputs in the basolateral amygdala, extinction of fear responses is believed to depend on a wider synaptic network including the ITCs (Likhtik et al. 2008). Low- and high-frequency stimulation of basolateral amygdala afferents was shown to induce long-term depression (LTD) and LTP in ITC neurons, respectively. Induction of LTP and LTD was NMDA dependent as it was prevented by the application of an NMDA antagonist (Royer and Paré 2002). NMDA receptors are heteromeric channels composed of the essential NMDA-type glutamate receptor subunit 1 (NR1) and one or more NMDA-type glutamate receptor subunits 2 (NR2), which determine channel kinetics (Paoletti and Neyton 2007). In particular, NMDA receptors containing NR2B subunits were shown to promote induction of synaptic plasticity (Malenka and Nicoll 1999).

In this study, we first analyzed the distribution and precise subcellular localization of Kv4.2 in the rodent amygdala focusing on ITC neurons. We then compared the subcellular arrangement of Kv4.2 subunits in ITC neurons to that in other types of central neurons, namely hippocampal pyramidal and cerebellar granule cells, to investigate whether Kv4.2 subunits feature a common organizational principle in their distribution. Finally, we studied whether gene-targeted deletion of Kv4.2 has an influence on NMDA receptors at ITC excitatory synapses.

## Materials and methods

### Materials

Paraformaldehyde, osmium tetroxide, uranyl acetate and pioloform were obtained from Agar Scientific Ltd. (Stansted, UK). EM grade glutaraldehyde was purchased from Polysciences Inc. (Warrington, PA). Thiopental was from Sandoz (Kundl, Austria). Lead (II) citrate was from Merck KGaA (Darmstadt, Germany), picric acid from Fluka GmbH (Buchs, Switzerland), and glycerin and sodium lauryl sulfate (SDS) from Carl Roth GmbH (Karlsruhe, Germany). Normal goat serum was from Bender (Vienna, Austria) and bovine serum albumin from Serva (Heidelberg, Germany). Gold-conjugated immunoglobulins were purchased from British BioCell Int. (Cardiff, UK). Cy™ 3-conjugated immunoglobulins were from Jackson ImmunoResearch Laboratories, Inc. (USA); Alexa Fluor^®^ 488-conjugated immunoglobulins were from Invitrogen™ (Paisley, UK). Biotinylated immunoglobulins, avidin-biotinylated horseradish peroxidase complex (Vectastain^®^) and Vectashield^®^ were from Vector Laboratories, Inc. (Burlingame, CA), Eukitt was from Kindler (Freiburg, Germany). Platinum and carbon rods were from Bal-Tec (Balzers, Liechtenstein). All remaining chemicals were from Sigma.

### Antibody specification

#### Mouse anti-Kv4.2 (209–225)

A mouse monoclonal anti-Kv4.2 antibody, NeuroMab clone K57/1 (mouse strain, Balb/C; myeloma cell, SP2/0), was obtained from the UC Davis/NIH NeuroMab Facility (Davis, CA). The antibody was produced against a synthetic peptide covering the amino acid sequence 209–225 (S1–S2 loop) of the rat Kv4.2 protein (100 % identity in rat and mouse). The antibody recognized a single band of approximately 72 kDa in immunoblot analysis of both rat and mouse brain membranes (Dabrowska and Rainnie 2010; Rhodes et al. 2004). The antibody was applied both in light and electron microscopy in the present study. The specificity of immunoreaction was tested and confirmed on tissue samples from Kv4.2^−/−^ mice (deletion of the KCND2 locus by homologous recombination; Guo et al. 2005).

#### Rabbit anti-Kv4.2 (454–469)

An affinity-purified anti-Kv4.2 antibody was obtained from Alomone Labs Ltd. (Jerusalem, Israel). This immune serum was raised in rabbit using immunogenic peptides corresponding to amino acid residues 454–469 of rat Kv4.2 (C-terminal domain; 15/16 residues identical in rat and mouse). Antibodies were affinity purified from immune sera on immobilized peptide and characterized by immunoblot analysis of rat brain membranes revealing a single band of approximately 72 kDa (Dabrowska and Rainnie 2010; Rhodes et al. 2004). Specificity of the antibodies in immunolabeling experiments was tested and confirmed on samples from Kv4.2^−/−^ mice.

### Neuronal marker proteins

#### μ-Opioid receptor (MOR)

The MOR immune serum was obtained from ImmunoStar (Hudson, WI). The serum was raised in rabbit using a synthetic peptide corresponding to amino acids 384–398 of rat MOR-1 (100 % identity in rat and mouse) coupled to bovine thyroglobulin with glutaraldehyde. The specificity of the antiserum was determined by immunlabeling of transfected cells, Western blot analysis and immunoisolation studies. Immunoblot analysis of rat brain membranes revealed a single band with a molecular weight of 67–72 kDa (Arvidsson et al. 1995).

#### Postsynaptic density protein 95 (PSD-95)

The PSD-95 antibody, clone 7E3-1B8 (Cat MAB1598, Lot NG1780754), was obtained from Millipore (Temecula, CA). The purified mouse monoclonal IgG_2a_ reacted with recombinant rat PSD-95 and identified a band at approximately 100 kDa on immunoblots of mouse and rat brain membranes.

#### NMDA-type glutamate receptor subunit 1 (NR1)

The NR1 antibody (Cat MAB363, Lot LV1387188) was obtained from Millipore (Temecula, CA). The purified mouse monoclonal IgG_2a_ recognized an epitope between amino acids 660–811 of the NR1 C-terminal domain and showed no cross reactivity with other NMDA-type glutamate receptors.

#### NMDA-type glutamate receptor subunit 2B (NR2B)

The NR2B antibody (code GluRe2C-Rb-Af264-1) was obtained from Frontier Science Co. Ltd (Hokkaido, Japan). The immune serum was raised in rabbit using a synthetic peptide corresponding to amino acids 1301–1456 of mouse NR2B, C-terminal domain. It identified a single band at approximately 180 kDa on immunoblots of mouse and rat brain membranes, with no cross reactivity to other ionotropic glutamate receptor subunits.

Specificity of the antibodies in immunolabeling experiments was control tested omitting the primary antiserum and applying the full set of secondary antibodies, and pre-adsorbing the immune serum with excess of the immunogenic peptides (concentration 10 μg/ml).

### Animals and tissue preparation

Morphological investigations were performed on adult male Sprague–Dawley rats (8–10 weeks; Department of Laboratory Animals and Genetics, Medical University, Vienna, Austria), adult male C57Bl/6 mice (10–12 weeks; Medical University Vienna, Austria) and adult male Kv4.2^−/−^ mice (10–12 weeks; J.M. Nerbonne, Washington University Medical School, St. Louis, MO). All experimental protocols were approved by the Austrian Animal Experimentation Ethics Board (Tierversuchsgesetz BGBl. 501/1989) in compliance with both, the European Convention for the Protection of Vertebrate Animals used for Experimental and Other Scientific Purposes (ETS no. 123) and the European Communities Council Directive (86/609/EEC). The authors further attest that all efforts were made to minimize the number of animals used and their suffering. Animals were deeply anesthetized by intraperitoneal injection of thiopental (12 mg/100 g body weight) and perfused transcardially with phosphate-buffered saline (PBS; 25 mM, 0.9 % NaCl, pH 7.4) followed by chilled fixative (respective buffer conditions are given beneath for the different techniques used). After fixation, brains were immediately removed from the skull, washed in 0.1 M PB and stored in 0.1 M PB containing 0.05 % sodium azide at 6 °C until processed further.

### Immunohistochemistry for light microscopy

Brains of animals (Sprague–Dawley rats, *n* = 4; C57Bl/6 mice, *n* = 4; Kv4.2^−/−^ mice, *n* = 2) were perfusion fixed with phosphate buffer (PB; 0.1 M, pH 7.4) containing 4 % formaldehyde and 15 % of a saturated solution of picric acid. Serial sections were sliced at 40 μm with a Vibroslicer (Leica Microsystems VT1000S; Vienna, Austria), placed in Tris-buffered saline (TBS; 50 mM, 0.9 % NaCl, pH 7.4) and processed according to the avidin-biotin peroxidase complex (ABC) method for free-floating sections. Briefly, sections were rinsed in TBS containing 0.3 % Triton X-100 (TBS-T; pH 7.4) over a period of 3 h. Endogenous peroxidase activity was blocked with 0.9 % H_2_O_2_ in TBS for 30 min and sections were rinsed in TBS-T (three times, 10 min each). After incubation with 10 % normal goat serum (NGS) plus 2 % bovine serum albumin (BSA) in TBS-T for blocking nonspecific binding sites, sections were incubated with primary antibodies diluted in TBS-T containing 2 % BSA for 38 h at 6 °C; dilution of mouse anti-Kv4.2_(209–225)_ was 1.25 μg/ml; dilution of rabbit anti-Kv4.2_(454–469_) was 0.8 μg/ml. After rinsing in TBS-T (three times, 10 min each), biotinylated secondary antibodies raised in goat were applied (1/800 in TBS-T containing 2 % BSA) for 90 min at room temperature (RT), rinsed in TBS-T again (three times, 10 min each) and treated with an avidin-biotinylated horseradish peroxidase complex (1/100 in TBS-T containing 0.2 % BSA) for 90 min at RT. Sections were reacted with 0.05 % 3,3′-diaminobenzidine (DAB) and 0.003 % H_2_O_2_ in TBS for 5–6 min, rinsed in TBS, mounted on chromalun gelatin-coated glass slides, air dried, dehydrated and covered with Eukitt^®^. Specificity of Kv4.2 immunostaining was controlled applying the antibodies on tissue samples from Kv4.2^−/−^ mice.

Analysis was performed under an Axiophot light microscope equipped with Plan-Neofluar and Plan-Apo objective lenses (Zeiss, Jena, Germany). Images were taken with an AxioCam (Zeiss), and whole images were level adjusted, sharpened, and cropped in Photoshop (Adobe) without changing any specific features.

For double-labeling experiments, an indirect immunofluorescence method was applied. Samples were prepared as described above for immunoperoxidase labelings. After blocking nonspecific binding sites with 10 % NGS and 2 % BSA in TBS-T, sections were incubated with a mixture of mouse anti-Kv4.2_(209–225)_ (2 μg/ml) and rabbit anti-MOR (immune serum, 1/10,000) for 48 h at 6 °C. Afterward, sections were rinsed in TBS-T and fluorophore-labeled secondary antibodies were applied in different combinations: Cy™ 3-donkey anti-rabbit (1:400) and Alexa Fluor^®^ 488 donkey anti-mouse (1:500); Alexa Fluor^®^ 488 donkey anti-rabbit (1:500) and Cy™ 3-donkey anti-mouse (1:400). All antisera were diluted in TBS-T containing 0.1 % BSA. The sections were embedded in Vectashield^®^ and analyzed under an AxioImager M1 epifluorescence microscope (Carl Zeiss Inc., Jena, Germany) equipped with EC Plan-Neofluar objective lenses (Carl Zeiss, Inc.) and following filter blocks: Alexa 488/Cy2 (excitation filter BP 480/40 nm; reflection short-pass filter 505 nm; emission filter BP 527/30 nm) and Cy3 (excitation filter BP 545/25 nm; reflection short-pass filter 570 nm; emission filter BP 605/70 nm). Images were displayed using the Openlab software (version 5.5.0; Improvision, Coventry, UK) linked to an Orca-ER CCD camera (Hamamatsu Corporation, Japan). Whole images were level adjusted, sharpened and cropped in Photoshop (Adobe) without changing any specific features.

### Pre-embedding immunoelectron microscopy

Brains of animals (Sprague–Dawley rats, *n* = 3; C57Bl/6 mice, *n* = 3; Kv4.2^−/−^ mice, *n* = 2) were perfusion fixed with PB containing 4 % formaldehyde, 0.05 % glutaraldehyde and 15 % of a saturated solution of picric acid. Tissue blocks from the forebrain comprising the amygdala were dissected and coronal sections were sliced with a Vibroslicer (Leica Microsystems VT1000S) at 70 μm thickness. Pre-embedding immunoperoxidase labeling for electron microscopy was performed as described previously (Sailer et al. 2006). Briefly, sections were incubated with increasing gradients of sucrose (5, 10 and 20 %) in PB at 6 °C, flash frozen on liquid nitrogen and rapidly thawed in PB to increase penetration of reagents. Sections were then incubated in 50 mM glycine in TBS for 1 h at RT for quenching of free aldehyde groups, followed by incubation in 10 % NGS and 2 % BSA in TBS for 2 h at RT for blocking of nonspecific binding sites. Primary antibodies were then applied in TBS containing 2 % BSA for 48 h at 6 °C; dilution of antibodies: anti-Kv4.2_(209–225)_ at 2.5 μg/ml; anti-Kv4.2_(454–469)_ at 1.6 μg/ml; MOR (immune serum) at 1/5,000. After washing in TBS, biotinylated secondary antibodies (Vector Laboratories Inc.) were applied (1:200 in TBS containing 2 % BSA, for 24 h at 6 °C). Sections were then treated with an avidin-biotinylated horseradish peroxidase complex (1:100 in TBS for 3 h at RT). The sections were reacted with 0.05 % DAB and 0.003 % H_2_O_2_ in TB for 6–7 min at RT and washed in TB. Sections were contrast enhanced by means of 2 % osmium tetroxide in PB (for 40 min at RT in the dark) and 1 % uranyl acetate in 50 % ethanol (for 30 min at RT in the dark) and embedded in epoxy resin (Durcupan ACM) on greased glass slides. Regions of interest were dissected and re-embedded in epoxy resin. Serial ultrathin sections (70–80 nm) were sliced with an ultramicrotome (Leica Microsystems VT1000S) and collected on formvar-coated copper slot grids.

Alternatively, a pre-embedding immunometal labeling was performed. After application of primary antibodies, sections were incubated with Nanogold^®^ conjugated Fab’ fragments (1:100 in TBS containing 2 % BSA, for 24 h at 6 °C). Nanogold particles were amplified with silver using the HQ Silver™ Enhancement kit (Nanoprobes Inc.) for 5–7 min at RT under light microscopy control. Sections were washed in MilliQ water and postfixed in 2 % glutaraldehyde in TB for 10 min at RT prior to embedding in epoxy resin. For double-labeling experiments, one of the primary antibodies was visualized by the immunometal reaction and the other was visualized by the immunoperoxidase reaction. Immunometal labeling was always carried out first. Ultrathin sections were examined in a Philips CM120 TEM, equipped with a Morada CCD camera (Soft Imaging Systems, Muenster, Germany). Whole images were level adjusted, sharpened and cropped in Photoshop (Adobe) without changing any specific feature.

#### Sampling and analysis of pre-embedding data

Subcellular compartments were considered immunopositive when the reaction product was visible at respective domains in at least two consecutive sections. Identification of synaptic profiles was based on the presence of synaptic vesicles accumulated in the presynaptic active zone, a distinct postsynaptic density and an identifiable synaptic cleft. Four vibratome sections per animal were immunostained, and two blocks each from the left and right amygdala were analyzed per section.

### SDS-digested freeze-fracture replica labeling (SDS-FRL)

SDS-FRL was performed with some modifications to the original method (Fujimoto 1995). Brains of animals (Sprague–Dawley rats, *n* = 4; C57Bl/6 mice, *n* = 4; Kv4.2^−/−^ mice, *n* = 2) were perfusion fixed with PB (0.1 M, pH 7.4) containing 1 % formaldehyde and 15 % of a saturated solution of picric acid. Forebrains were cut into 140 μm thick coronal sections with a Vibroslicer (Leica Microsystems VT1000S) from where samples were dissected of respective brain areas and cryoprotected with 30 % glycerol in 0.1 M PB overnight at 6 °C. Samples were then frozen by use of a high-pressure freezing machine (HPM 010; Bal-Tec, Balzers, Liechtenstein) and fractured by a double-replica method in a freeze-etching device (BAF 060; Bal-Tec). Fractured faces were replicated by evaporation of carbon (rotating) by means of an electron beam gun positioned at a 90° angle to a thickness of 5 nm and shadowed unidirectionally with platinum–carbon at a 60° angle (thickness 2 nm). Finally, a 15 nm thick layer of carbon was applied from a 90° angle (rotating). Tissue was solubilized in a solution containing 2.5 % sodium lauryl sulfate (SDS) and 20 % sucrose made up in 15 mM Tris buffer, pH 8.3, on a shaking platform for 18 h at 80 °C. Replicas were kept in the same solution at RT until processed further.

On the day of immunolabeling, replicas were washed in TBS containing 0.05 % BSA and incubated in a blocking solution containing 5 % BSA in TBS for 1 h at RT. Subsequently, the replicas were incubated in the primary antibody diluted in TBS containing 2 % BSA and 2 % NGS, overnight at 6 °C. Dilution of antibodies used was: anti-Kv4.2_(209–225)_ at 2.5 μg/ml; anti-Kv4.2_(454–469)_ at 1.6 μg/ml; MOR (immune serum) at 1/5,000. After several washes in TBS, the replicas were reacted with gold-conjugated secondary antibodies (British BioCell Int.) made up in TBS (1:30) containing 2 % BSA overnight at 6 °C. They were then washed in MilliQ water, mounted on formvar-coated 100-line copper grids and analyzed in a Philips CM120 TEM equipped with a Morada CCD camera (Soft Imaging Systems). Whole images were level adjusted, sharpened and cropped in Photoshop (Adobe) without changing any specific features. The specificity of the immunolabeling was controlled and confirmed (1) applying the respective pre-immune serum, (2) omitting primary antibodies with following application of the full set of secondary antibodies and (3) applying antibodies on samples from Kv4.2^−/−^ mice, respectively.

#### Sampling and analysis of SDS-FRL data

Four to seven replicas were used for quantification of Kv4.2 immunolabeling in the following brain areas: ITCs, field CA1 of hippocampus (mouse, 1.4–2.0 mm caudal to Bregma; rat, 3.1–3.8 mm caudal to Bregma) and the cerebellar cortex (mouse, 6.0–6.6 mm caudal to Bregma; rat, 11.3–12.3 mm caudal to Bregma). Subcellular profiles were selected at random within a cell and within each area. Electron micrographs of such profiles were taken at a magnification of 43*k*×–110*k*×. The magnification was verified by use of a calibration grid. Quantification was performed using the software iTEM CE (Olympus Soft Imaging Solutions) and data were expressed as mean ± SD. To establish the frequency of NR1 synapses containing the NR2B subunit in wild-type and Kv4.2^−/−^ mice, glutamatergic synapses were selected at random over the entire dendritic tree of ITC neurons from proximal to distal sites. To compare the frequency of such synapses in wild-type versus Kv4.2^−/−^ mice, the Chi-square test (*p* = 0.05) was applied. For comparing immunoparticle densities in different neuronal domains, Mann–Whitney *U* (*p* = 0.05) was used. Statistical analysis was carried out in Prism (GraphPad).

#### Nearest neighbor distance analysis

Images were analyzed using FIJI software (distributed under the General Public License, GPL), and distance measurements were done using macros written for Excel (Microsoft). Immunogold particles were selected by automatic tracking of maximal grey levels in two-dimensional grey-scale images followed by visual inspection to discriminate between actual gold particles and background. The *xy*-coordinates of each particle were then extracted, the distance between particles measured and the smallest value taken as the nearest neighbor distance (NND). NNDs for all particles were tabulated and presented in cumulative frequency plots for each of the subcellular domains investigated. For the identification of tight clusters of immunogold particles, binary images were created with immunogold particles depicted as single-pixel dots and a circle (radius = 20 nm) drawn around each particle using the minimum filter function in FIJI. Immunogold particles were considered to form tight clusters when these circles overlapped. For establishing the overall distribution pattern of immunogold particles, the centroids of overlapping circles were defined in the respective binary images, and NNDs between these centroids and the remaining scattered particles were determined. For computing random distributions, centroids of tight clusters and scattered particle locations were re-distributed randomly over an area with the same size as the average of the sampled profiles, and 100 such random distributions were created for each subcellular domain. For comparing NNDs in different domains, Mann–Whitney *U* test (*p* = 0.05) was used. Statistical analysis was carried out in SPSS (IBM).

## Results

### Non-uniform distribution of Kv4.2 in the rodent amygdala

**Fig. 1:**
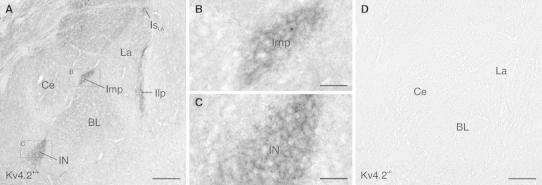
Distribution of Kv4.2 in the mouse amygdala revealed by immunoperoxidase labeling. **a** Prominent Kv4.2 immunoreactivity is detected in all ITC clusters and the ITC nucleus in C57Bl/6 wild-type mice (Kv4.2^+/+^). Only low to moderate levels of immunoreactivity are observed in other amygdaloid areas than the ITC. **b** Higher magnification of the Imp cluster (*boxed* area in **a**). Diffuse Kv4.2 immunoreactivity is primarily observed in the neuropil. **c** A similar immunostaining pattern for Kv4.2 is observed in the ITC nucleus (*boxed* area in **a**). **d** Specificity of Kv4.2 immunolabeling is confirmed on respective brain areas from a Kv4.2^−/−^ mouse. *BL* basolateral amygala, *Ce* central nucleus of amygdala, *Ilp* lateral paracapsular ITC cluster, *Imp* medial paracapsular ITC cluster, *IN* ITC nucleus, *Is*_*LA*_ supralateral ITC cluster, *La* lateral amygdala. *Scale bar* 200 μm (**a**, **d**); 40 μm (**b**, **c**)

**Fig. 2:**
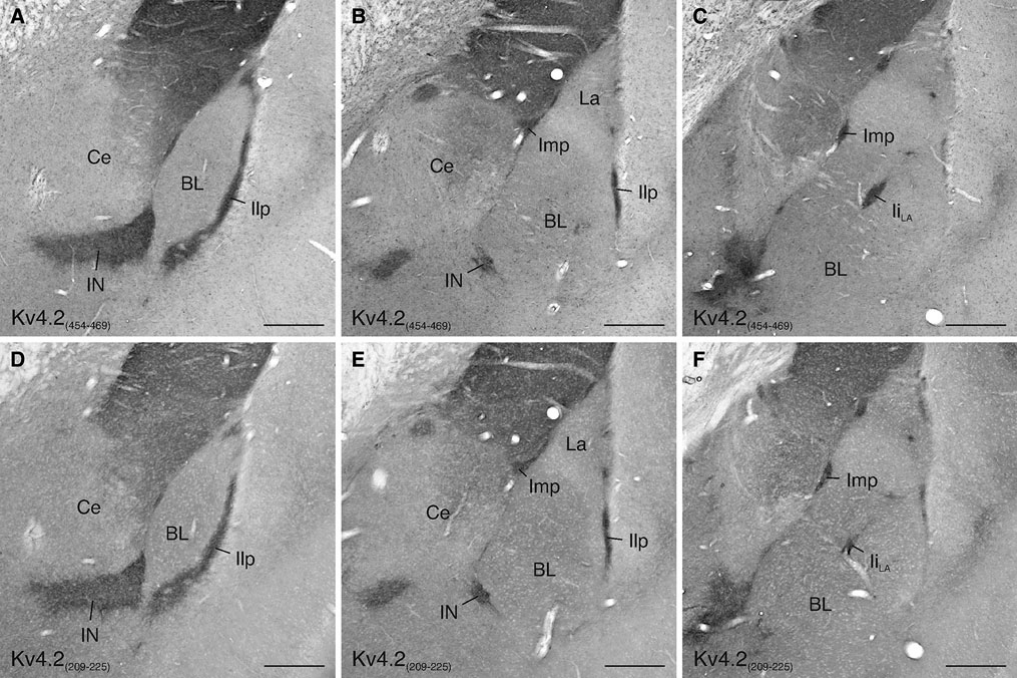
Kv4.2 immunolabeling in the rat amygdala using antibodies directed against different regions of the Kv4.2 protein. **a**–**c** The Kv4.2_(454–469)_ antibody reveals prominent immunolabeling of all ITC clusters and the ITC nucleus from rostral (**a**) to medial (**b**) and caudal (**c**) levels of the amygdala using an immunoperoxidase staining technique. Other amygdaloid areas are only faintly labeled. **d**–**f** An equivalent immunolabeling pattern is observed with the Kv4.2_(209–225)_ antibody. *BL* basolateral amygdala, *Ce* central nucleus of amygdala, *Ii*_*LA*_ intralateral paracapsular ITC cluster, *Ilp* lateral paracapsular ITC cluster, *Imp* medial paracapsular ITC cluster, *IN* ITC nucleus, *La* lateral nucleus of amygdala. *Scale bar* 350 μm (**a**–**f**)

The localization of Kv4.2 subunits was analyzed in the amygdaloid complex of mouse and rat brain using two antibodies directed against different recognition sites of the Kv4.2 protein (amino acids 209–225 and 454–469, respectively). The same distribution pattern was observed for both species and both antibodies. In the mouse brain (Fig. 1), prominent Kv4.2 subunit immunoreactivity was detected in all subdivisions of the ITCs described so far (Busti et al. 2011), and all were labeled to a similar extent. Very low to moderate levels of Kv4.2 subunit immunoreactivity were observed in other amygdaloid areas such as the lateral, basal and central nucleus of the amygdala. These areas were homogeneously immunolabeled, albeit at a slightly different density, and the staining patterns appeared diffuse (Fig. 1a). When analyzing Kv4.2 immunolabeling in the ITCs at higher resolution, it appeared enriched in the neuropil and sparse in cell bodies (Fig. 1b, c). However, distinct axonal fibers, dendrites or glial elements could not be differentiated in light microscopy. Strongly immunopositive puncta were not observed and all neurons within a cluster seemed to be labeled, not only a subpopulation of cells. Specificity of immunolabeling was tested and confirmed on samples from Kv4.2^−/−^ mice (Fig. 1d). The same distribution pattern as in mouse brain was observed for Kv4.2 subunit labeling in rat brain, and both antibodies used (Kv4.2_454–469_, Fig. 2a–c; Kv4.2_209–225_, Fig. 2d–f) gave identical results. All ITCs were strongly immunoreactive, and the prominent labeling of these clusters stood out against the faint labeling of other amygdaloid areas (Fig. 2a–f).

**Fig. 3:**
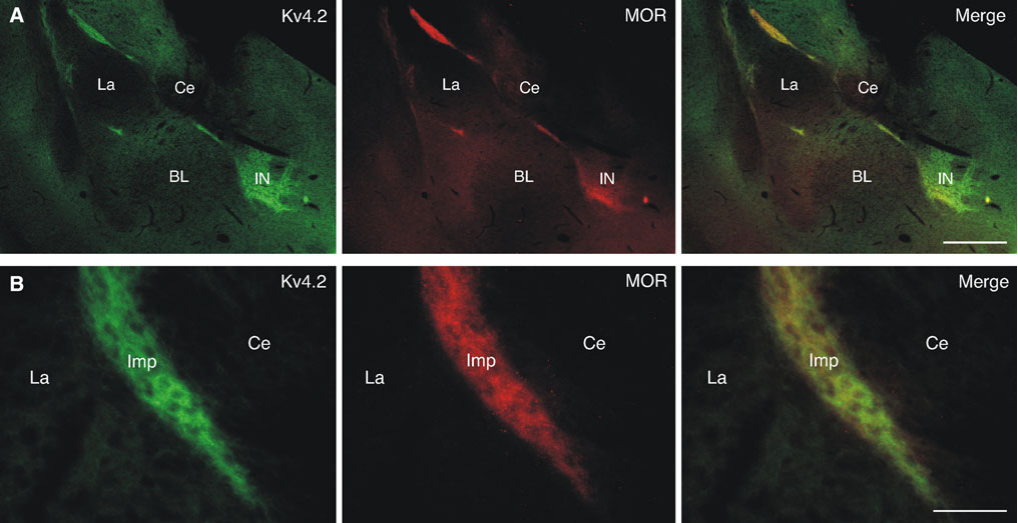
Colocalization of Kv4.2 and μ-opioid receptors (MOR) in the ITCs. **a** Double-labeling immunofluorescence for Kv4.2 (in *green*) and MOR (in *red*) reveals a high degree of coexistence in the amygdala, which is evident in the merged images. **b** At higher resolution, immunoreactivity for Kv4.2 (in *green*) as well as for MOR (in *red*) appears dense and diffuse in ITC clusters such as the Imp. The distribution profile of these proteins is highly similar (*merge*). *BL* basolateral amygdala, *Ce* central nucleus of amygdala, *Imp* medial paracapsular ITC cluster, *IN* ITC nucleus, *La* lateral amygdala. *Scale bar* 350 μm (**a**), 50 μm (**b**)

To confirm a distinct localization of Kv4.2 subunits to ITC neurons, we performed double-labeling immunofluorescence experiments applying antibodies against Kv4.2 (Kv4.2_209–225_) and μ-opioid receptors (MOR), a receptor highly enriched in ITC neurons (Likhtik et al. 2008). All ITC subdivisions were consistently and densely co-labeled for Kv4.2 subunits and MOR, both in mouse and in rat amygdala (Fig. 3).

### Subcellular localization of Kv4.2 subunits to somato-dendritic domains of ITC neurons

**Fig. 4:**
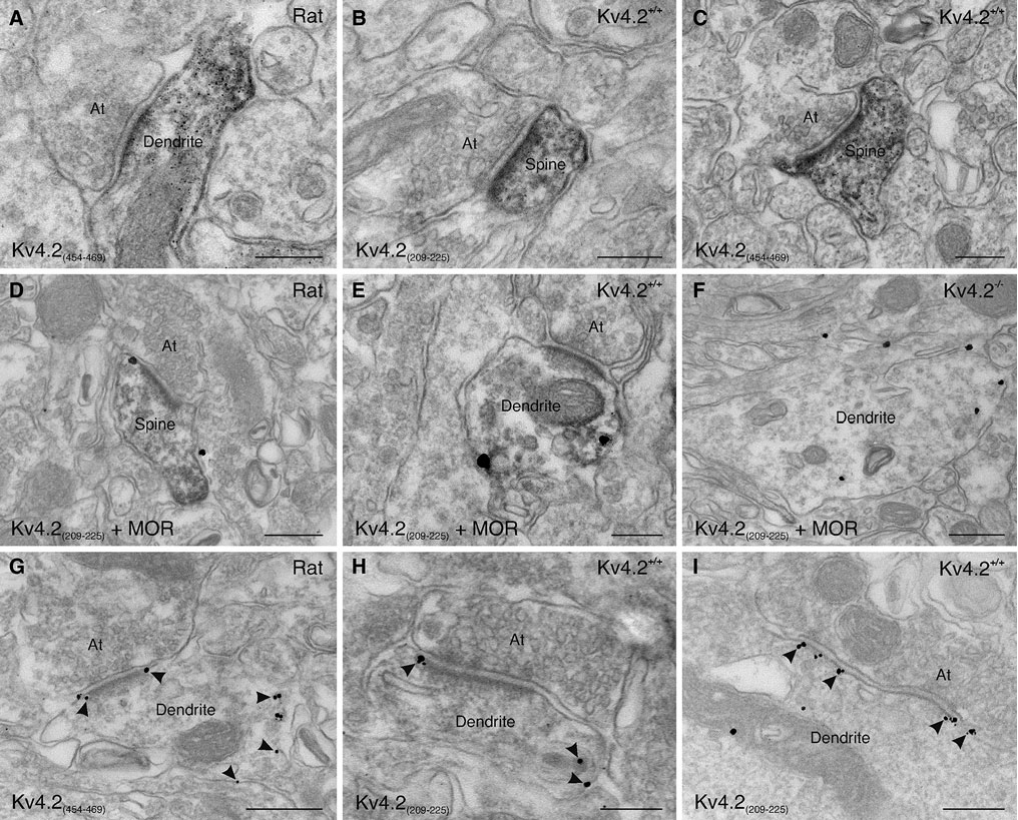
Subcellular localization of Kv4.2 to somato-dendritic domains of ITC neurons revealed by pre-embedding immunoperoxidase and immunometal electron microscopy. **a** A small dendritic trunk of a neuron in the rat ITC nucleus displays dense and diffuse immunolabeling for Kv4.2 [Kv4.2_(454–469)_ antibody]. The immunolabeling appears as an electron-opaque reaction product. A synaptic terminal, contacting this dendrite, is free of any immunolabeling. **b** A dendritic spine of a neuron in the mouse ITC nucleus shows dense and diffuse labeling for Kv4.2 [Kv4.2_(209–225)_ antibody]. An axon terminal contacting this spine, as well as other cellular profiles, is free of any immunolabeling. **c** A dendritic spine of a mouse Imp neuron shows the same dense and diffuse labeling pattern for Kv4.2 [Kv4.2_(454–469)_ antibody]. **d** In a double-labeling experiment, a Kv4.2 immunopositive spine of a rat Imp neuron (immunoperoxidase reaction) is also immunoreactive for MOR (immunometal reaction). **e** A small dendrite of a mouse Imp neuron, immunolabeled for Kv4.2 (immunoperoxidase reaction), is also immunoreactive for MOR (immunometal reaction). An axon terminal, contacting this dendrite, is free of any immunolabeling. **f** In a sample from a Kv4.2^−/−^ mouse, a large dendrite of an Imp neuron is labeled for MOR (immunometal reaction), but devoid of Kv4.2 immunolabeling (immunoperoxidase reaction). **g** Immunometal particles (indicated by *arrowheads*) for Kv4.2, when applying the Kv4.2_(454–469)_ antibody, are observed at the intracellular side of the plasma membrane of a rat Imp neuron. The postsynaptic specialization of an excitatory synapse is free of any immunolabeling. Some immunoparticles appear localized perisynaptically. **h** Immunometal particles (indicated by arrowheads) for Kv4.2 appear at the intracellular side of an ITC neuron dendrite also when applying the Kv4.2_(209–225)_ antibody. Both the axon forming a synapse with this dendrite and the postsynaptic specialization are free of any immunolabeling. **i** The plasma membrane of an Imp dendrite is decorated with immunoparticles at the intracellular side applying the Kv4.2_(209–225)_ antibody. The postsynaptic specialization of an inhibitory synapse on this dendrite is also free of any immunolabeling.* At* axon terminal,* MOR* μ-opioid receptor. *Scale bar* 200 nm (**a**–**c**, **e**, **h**, **i**); 300 nm (**d**, **f**, **g**)

Light microscopic analysis strongly suggest that Kv4.2 subunits are expressed in neuronal processes and absent in glial elements. To analyze the precise subcellular localization of Kv4.2 subunits, we performed pre-embedding immunoelectron microscopy (Fig. 4). Immunoreaction for MOR was used again in double-labeling experiments for the identification of ITC profiles (Fig. 4d–f). By means of the pre-embedding immunoperoxidase technique, Kv4.2 immunoreactivity was observed in dendrites and spines of ITC neurons both in rat (Fig. 4a, d) and in mouse amygdala (Fig. 4b, c, e). These neuronal elements appeared homogeneously labeled without distinct clustering of the reaction product at specific subdomains. Density of immunolabeling in the soma of ITC neurons was moderate, and axonal profiles were immunonegative including the axon initial segment, the axon trunk and terminals. Glial cells appeared also immunonegative. Specificity of Kv4.2 immunoreaction was verified in samples from Kv4.2^−/−^ mice (Fig. 4f). Noteworthy, for both the Kv4.2_(209–225)_ and the Kv4.2_(454–469)_ antibodies, the electron-opaque reaction product was detected at the intracellular, protoplasmic side of ITC neurons. Such staining pattern was expected for the Kv4.2_(454–469)_ antibody, since it was directed against an intracellular C-terminal domain of the protein. However, it was unexpected for the Kv4.2_(209–225)_ antibody, as it was raised against an extracellular epitope of Kv4.2 (S1–S2 loop). As immunoperoxidase reaction products diffuse within the cytoplasm, which hampers a precise subcellular localization of the epitope, we further analyzed Kv4.2 localization by means of the pre-embedding immunometal (nanogold/silver) technique in another set of experiments (Fig. 4g–i). This approach yields a higher spatial resolution than the immunoperoxidase technique, although at the cost of antibody penetration and labeling sensitivity. For both the Kv4.2_(209–225)_ and Kv4.2_(454–469)_ antibodies, the reaction products were observed at the intracellular side of the plasma membrane. Rarely, immunometal particles were localized to intracellular organelles such as the endoplasmic reticulum. In line with the results of the immunoperoxidase technique, immunometal particles for Kv4.2 subunits were restricted to somato-dendritic domains of ITC neurons. Within ITC dendritic trunks, clustering of immunoreaction product was not observed, and the immunolabeling appeared homogeneous throughout the entire extent of dendrites including the head and neck of spines. Postsynaptic membrane specializations of symmetric (presumably inhibitory) as well as asymmetric (presumably excitatory) synapses were free of Kv4.2 immunolabeling (Fig. 4g–i). However, as various subcellular domains are differentially accessible for antibody binding using pre-embedding techniques—due to diffusion restrictions or steric hindrance—lack of immunolabeling does not necessarily imply absence of the antigen in this domain. This is in particular true for structures where the protein density is very high, such as postsynaptic membrane specializations. In order to access such protein-rich domains and to obtain semi-quantitative data on subunit density and arrangement, we next performed SDS-digested freeze-fracture replica immunolabeling (SDS-FRL). This technique facilitates the localization of integral membrane proteins beyond the limitations of thin-section electron microscopy and allows antibody binding without diffusion restrictions.

### Scattered distribution of Kv4.2 subunits in the somato-dendritic plasma membrane of ITC neurons

**Fig. 5:**
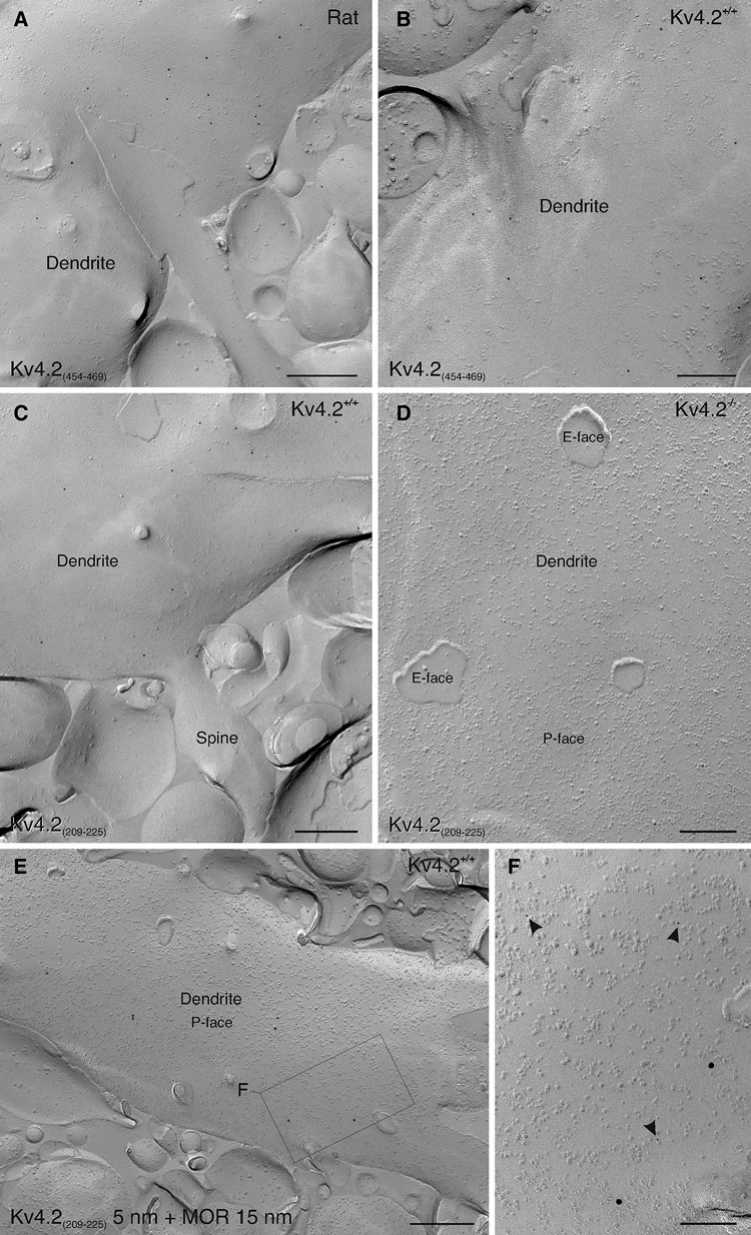
SDS-FRL confirms the localization of Kv4.2 in the somato-dendritic plasma membrane of ITC neurons. **a** Immunoparticles labeling Kv4.2 subunits (10 nm gold) are scattered on the plasma membrane of a large dendritic trunk of a rat ITC neuron. The immunolabeling is restricted to the plasma membrane P-face. **b** Scattered immunopartices are also observed in the plasma membrane P-face of a large dendritic trunk of a mouse ITC neuron. **c** In addition to the labeling of the dendritic trunk, Kv4.2 immunoparticles are localized to the head and neck of spines. **d** Both the plasma membrane E- and P-face of an ITC dendrite from a Kv4.2^−/−^ mouse are immunonegative when reacted with the Kv4.2_(209–225)_ antibody (10 nm gold). **e** An ITC dendritic trunk from a wild-type mouse (Kv4.2^+/+^) is immunopositive for both Kv4.2_(209–225)_ (5 nm gold) and MOR (15 nm gold) in a double-immunolabeling experiment. **f** At higher resolution (*boxed* area in **e**), both 5 and 15 nm gold particles revealing Kv4.2 subunits and MOR, respectively, are visible on the P-face of the dendritic plasma membrane. *MOR* μ-opioid receptor. *Scale bar* 300 nm (**a**, **c**); 200 nm (**b**, **d**); 400 nm (**e**); 150 nm (**f**)

On application of the Kv4.2_(209–225)_ and the Kv4.2_(454–469)_ antibodies in SDS-FRL (Fig. 5), a similar labeling pattern was observed as described in the previous sections. Kv4.2 immunogold particles were scattered all over the somato-dendritic plasma membrane of ITC neurons (Fig. 5a–c), although some aggregations of particles could be observed. This pattern was found both in rat (Fig. 5a) and in mouse ITC neurons (Fig. 5b, c). Noteworthy, labeling for both the Kv4.2_(209–225)_ and the Kv4.2_(454–469)_ antibodies appeared at the protoplasmic (P-) face of the membrane confirming our pre-embedding findings. Specificity of Kv4.2 immunolabeling was tested and confirmed again on tissue samples from Kv4.2^−/−^ mice (Fig. 5d). To positively identify ITC profiles, double staining of Kv4.2 and MOR was performed. Immunoparticles for MOR were scattered throughout the somato-dendritic plasma membrane (Fig. 5e), including the head and neck of spines. The distribution pattern of immunoparticles was homogeneous, and formation of distinct clusters was not observed. The density of MOR labeling appeared similar to the density of Kv4.2 labeling, and the double-immunostaining for Kv4.2 and MOR confirmed that the profile analyzed belonged to ITC neurons (Fig. 5e, f).

**Fig. 6:**
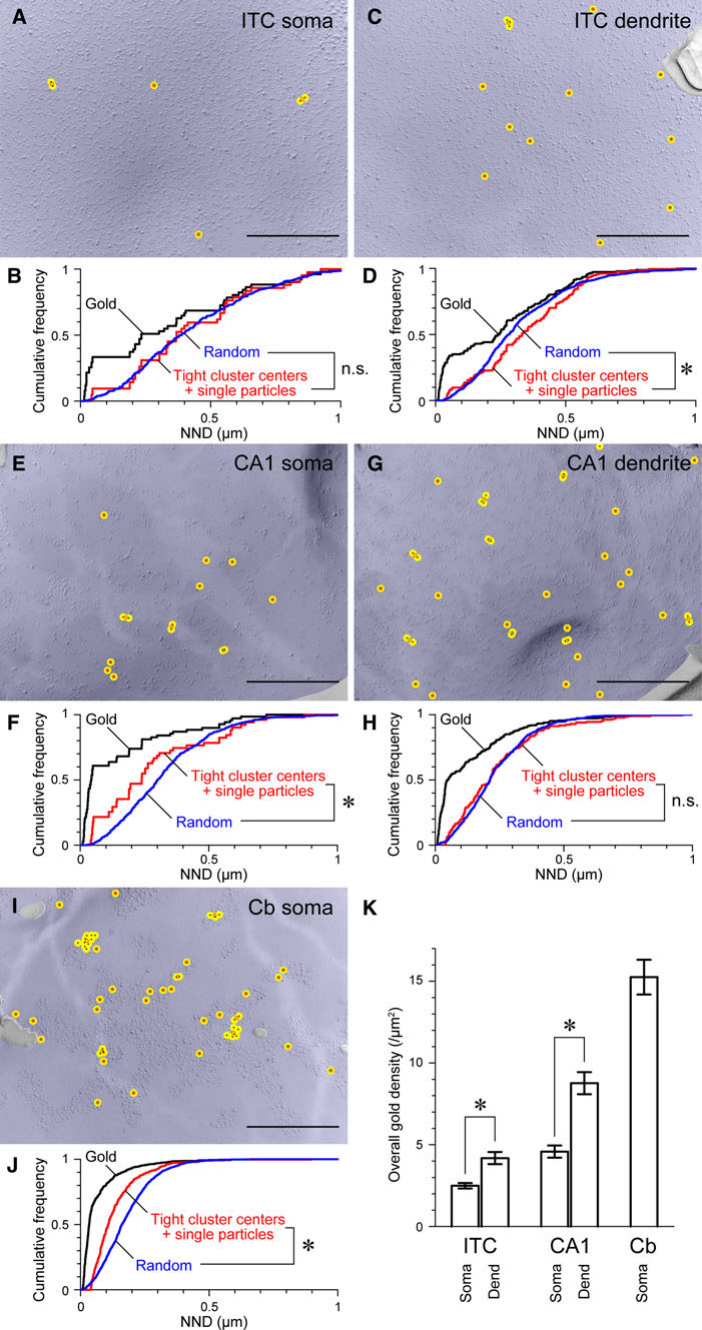
Quantitative characterization of the distribution of Kv4.2 immunogold particles in ITC neurons and other central principal cells. **a** A sample SDS-FRL image of a portion of an ITC neuron soma. The area of the relevant profile is colored in *blue*. Immunogold particles labeling Kv4.2 subunits are marked with a *black dot*, a 20-nm radius *circle* around each particle is shown in *yellow* and the centroid of overlapping *circles* is marked with a *red open circle*. A tight cluster of particles is defined as particles residing within the overlapping *yellow circles*. **b** Cumulative probability curves for the nearest neighbor distances (NNDs) between individual immunogold particles (in *black*) and tight cluster centers along with single particles (in *red*), and NNDs of the calculated random distribution (in *blue*). **c** A sample image of a portion of an ITC dendrite and **d** respective NND analysis. **e**–**h** Sample images and NND analyses for CA1 pyramidal neuron soma and dendrite. **i**–**j** Sample image and NND analysis for cerebellar granule cell soma. **k** Histogram showing the immunogold particle density in subcellular domains of ITC neurons (soma: 8 profiles with a mean area of 2.60 μm^2^; dendrite: 22 profiles with a mean area of 2.05 μm^2^), CA1 pyramidal neurons (soma: 6 profiles with a mean area of 2.52 μm^2^; dendrite: 19 profiles with a mean area of 1.52 μm^2^) and cerebellar granule cells (soma: 25 profiles with a mean area of 2.54 μm^2^). *Cb* cerebellar granule cell, *NND* nearest neighbor distance. *Error bars*, SEM. *p < 0.05, Mann–Whitney *U*. *Scale bar* 500 nm (**a**, **c**, **e**, **g**, **i**)

The Kv4.2_(454–469)_ antibody was then used to quantitatively characterize the Kv4.2 subunit distribution. The overall immunogold particle density on the plasma membrane was significantly higher in the dendritic compared to the somatic compartment of mouse ITC neurons (Fig. 6a, c) with 4.18 ± 1.75 particles/μm^2^ plasma membrane at the level of dendrites (profiles sampled = 22; total area analyzed = 45 μm^2^; Fig. 6k) and 2.49 ± 0.48 particles/μm^2^ plasma membrane in the soma (profiles sampled = 8; total area analyzed = 21 μm^2^; *p* = 0.00, Mann–Whitney *U*; Fig. 6k). Background labeling, established in respective profiles of Kv4.2^−/−^ mice, was 0.51 ± 0.29 particles per μm^2^ (area analyzed = 50 μm^2^). To describe the distribution pattern, nearest neighbor distances (NNDs) between the immunogold particles were calculated. In the cumulative distribution curves, a sharp rise from 0 to ~40 nm was found, indicating the presence of tight clusters of immunogold particles (Fig. 6b, d). The presence of doublets or triplets of particles within 40 nm from each other could often be observed (Fig. 6a, c). At present, we cannot resolve whether these tight clusters of gold particles represent labeling of different subunits composing the same tetrameric channel or whether they indicate the presence of several Kv4.2 containing channels tightly clustered. Such doublets or triplets were absent in immunolabelings of respective profiles in Kv4.2^−/−^ mice (Fig. 5d) indicating that the antibodies and the conjugated gold particles themselves were not aggregated. Further analysis was performed to understand whether the particles form distinct distribution patterns in addition to the tight clustering. A 20-nm radius circle was drawn around each particle and particles residing in the overlapping circles were defined as a tight cluster of particles. These tight clusters were replaced with a single dot at the centroid of overlapping circles (Fig. 6). NNDs were calculated and compared with the random distribution, which was created from 100 trials with the same number of particles (centroids of tight clusters plus single scattered particles) distributed over an area with the same size as the average of the sampled profiles. In the case of the ITC soma, the sampled and created random NND distributions completely overlapped (Fig. 6b; *p* = 0.80, Mann–Whitney *U*), whereas for the ITC dendrite, the distribution of the NND for sampled particles was shifted to the right compared to the random distribution (Fig. 6d; *p* = 0.03, Mann–Whitney *U*). This implies that Kv4.2 subunits are homogeneously distributed throughout the plasma membrane. Consistent with this idea, NNDs between the tight cluster centers along with the single particles in the ITC dendrite were not significantly different from those in the ITC soma (*p* = 0.09, Mann–Whitney *U*).

### Varying densities and distribution patterns of Kv4.2 subunits in different types of central principal neurons

To evaluate whether there is a common organizational principle in the distribution of Kv4.2 subunits in central neurons, we compared the Kv4.2 subunit distribution in ITC domains to that of other cell types. In CA1 pyramidal neurons, immunogold particles were also scattered in the somatic plasma membrane and throughout their entire dendritic tree (Fig. 6e, g), including the head and neck of spines. The overall immunogold particle density was significantly higher in dendrites (8.77 ± 2.93 particles/μm^2^; profiles sampled = 19; total area analyzed = 29 μm^2^) compared to the soma (4.58 ± 0.92 particles/μm^2^, profiles sampled = 6; total area analyzed = 15 μm^2^; *p* = 0.01, Mann–Whitney *U*), similar to ITC neurons (Fig. 6k). Tight clusters of immunoparticles with NNDs smaller than 40 nm were also apparent in the CA1 neurons. In the soma of CA1 neurons, NNDs between the tight cluster centers along with the single particles were smaller compared to the random distribution (Fig. 6f; *p* = 0.02, Mann–Whitney *U*), which indicates that Kv4.2 containing channels form loose clusters (smaller than a few hundred nanometers in diameter) as well as tight clusters (smaller than 40 nm in diameter). In CA1 dendrites on the other hand, the distribution of NND values for the sampled particles was highly similar to the random distribution (Fig. 6h; *p* = 0.50, Mann–Whitney *U*). These results indicate that the soma of CA1 pyramidal neurons tend to have loose clusters of Kv4.2 containing channels with large areas of the plasma membrane devoid of channels, whereas dendrites have a higher density, nearly twice as much as in the soma, but lacking a clear spatial arrangement.

In contrast to both ITC and CA1 pyramidal neurons, the presence of large and distinct particle aggregations was evident in the soma of cerebellar granule cells (Fig. 6i). In this type of cell, the overall particle density was much higher compared to ITC and CA1 pyramidal neurons (Fig. 6k; 15.25 ± 5.31 particles/μm^2^, profiles sampled = 25, total area analyzed = 64 μm^2^). The cumulative NND distribution was shifted to the left of the random distribution (Fig. 6j; *p* = 0.00, Mann–Whitney *U*), clearly indicating that Kv4.2 containing channels in the cerebellar granule cell soma are closely packed together in clusters.

### Kv4.2 channel subunits are localized to extrasynaptic sites

**Fig. 7:**
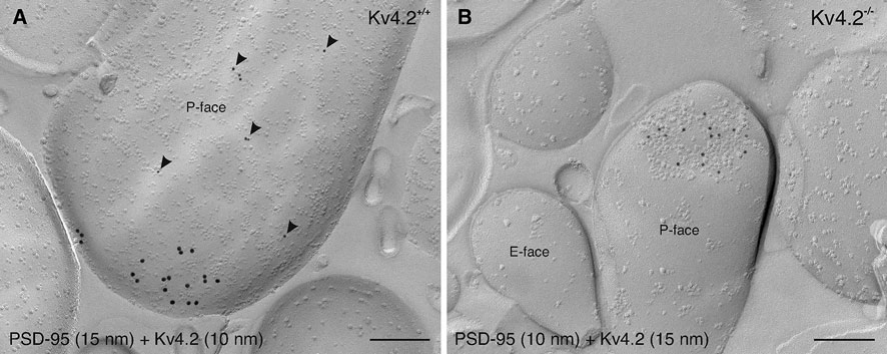
Segregation of Kv4.2 to extrasynaptic domains. **a** In a double-immunolabeling experiment for PSD-95, visualized with 15 nm gold particles, and Kv4.2, visualized with 10 nm gold particles (*arrowheads*), Kv4.2 subunits are observed at extrasynaptic sites of an ITC dendritic spine in a wild-type mouse (Kv4.2^+/+^). Conversely, PSD-95 labeling can be seen within the postsynaptic membrane specialization of an excitatory synapse. **b** In a Kv4.2^−/−^ mouse, labeling for PSD-95 (10 nm gold) can clearly be detected within the postsynaptic membrane specialization of a dendritic spine. No immunoreactivity for Kv4.2 (15 nm gold) is observed in Kv4.2^−/−^ mouse tissue. *Scale bar* 200 nm (**a**, **b**)

**Fig. 8:**
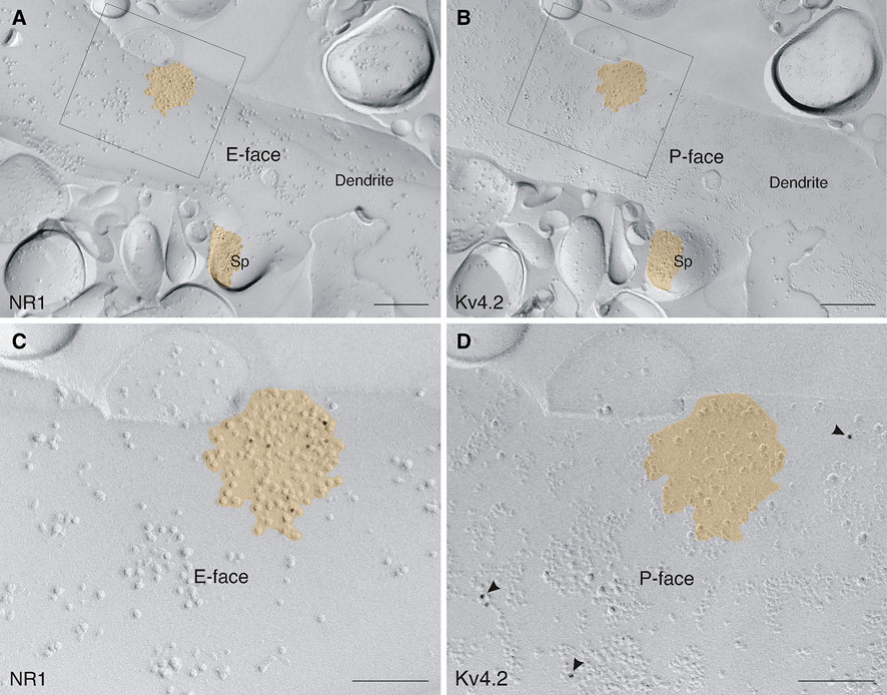
Kv4.2 does not reside within the postsynaptic membrane specializations of excitatory synapses as revealed by a double-replica approach.** a** Immunogold particles revealing NR1 subunits (10 nm gold) are localized to the E-face of an ITC dendrite.** b** On the mirror replica, immunogold particles revealing Kv4.2 subunits (10 nm gold) are observed on the P-face of the same dendrite. **c** At higher resolution (*boxed* area in **a**), immunolabeling for NR1 subunits is evident on the E-face of a postsynaptic membrane specialization of a glutamatergic synapse (shown in *orange*), which is characterized by the clustering of intramembrane particles. **d** Immunolabeling for Kv4.2 subunits is present on the P-face of the extrasynaptic plasma membrane at higher resolution (*boxed* area in **b**). The postsynaptic membrane specialization by itself is free of any Kv4.2 immunolabeling. *Sp* spine. *Scale bar* 300 nm (**a**, **b**); 150 nm (**c**, **d**)

To confirm our pre-embedding immunoelectron microscopy data—indicating a lack of association between Kv4.2 subunits and synaptic specializations—we carried out double-immuno SDS-FRL experiments for Kv4.2 subunits and the postsynaptic density protein 95 (PSD-95), which represents a P-face marker for excitatory synapses (Fig. 7). Indeed, immunolabeling for Kv4.2 subunits was never detected in asymmetric synapses identified by the PSD-95 labeling (Fig. 7a). However, PSD-95 is not an integral membrane protein and could easily be solubilized during the replica preparation process (SDS-digestion). Thus, not all excitatory synapses are probably recognized by the PSD-95 immunolabeling. To verify further the absence of Kv4.2 subunits in excitatory synapses, we studied the distribution of Kv4.2 subunits (labeling at the membrane P-face) and NMDA receptor type-1 subunits (NR1; labeling at the membrane E-face) using a double-replica approach, in which both faces of the same plasma membrane can be analyzed simultaneously (Fig. 8). NR1 was used as a marker for excitatory synapses since it is localized primarily to postsynaptic specializations of this type of synapse. The membrane area of a postsynaptic specialization was defined by demarcating the boundary of an intramembrane particle cluster on the E-face immunopositive for NR1 (Fig. 8a, c). This area was then projected onto the corresponding P-face and analyzed for Kv4.2 immunolabeling (Fig. 8b, d). By means of this approach, we confirmed that Kv4.2 subunits did not reside within synaptic specializations of excitatory synapses, but rather exclusively at extrasynaptic sites in ITC neurons.

### Kv4.2 gene-targeted deletion induces an increase in the frequency of NMDA synapses containing the NR2B subunit

**Fig. 9:**
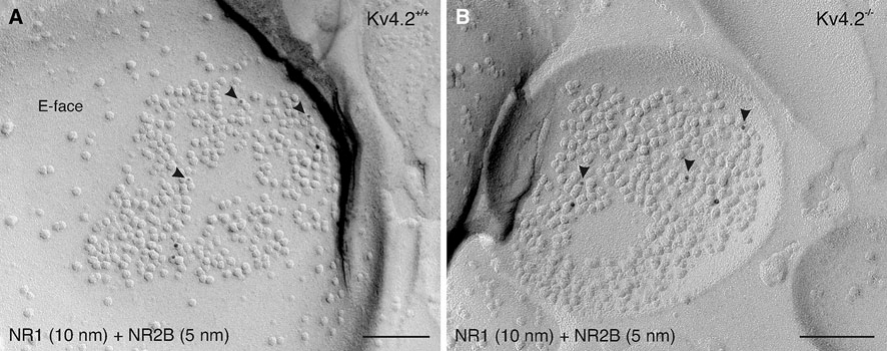
Modulatory action of Kv4.2 on the synaptic expression of NR2B. **a** In a double-immunolabeling experiment for NMDA-type glutamate receptor subunit 1 (NR1) and subunit 2B (NR2B), both NR1 (10 nm gold) and NR2B (5 nm gold; *arrowheads*) are observed on the P-face of the postsynaptic membrane specialization of an excitatory synapse in an ITC neuron from a Kv4.2^+/+^ mouse. **b** In an ITC neuron from a Kv4.2^−/−^ mouse, NR1 (10 nm gold) and NR2B immunolabeling (5 nm gold; *arrowheads*) are observed in the postsynaptic membrane of an excitatory synapse at a density similar to that observed in Kv4.2^+/+^ neurons. Yet, the frequency of such synapses, co-immunolabeled for both the NR1 and NR2B subunits, was increased in ITC neurons of Kv4.2^−/−^ mice. *Scale bar* 150 nm (**a**, **b**)

As it was reported that up- and down-regulation of Kv4.2 gene expression in transgenic animals induces a bidirectional modification of NR2 subunits in excitatory synapses with altered NR2B subunit levels (Jung et al. 2008), we analyzed the frequency of glutamatergic synapses possessing NMDA receptors and containing the NR2B subunit in wild-type animals (Fig. 9a) compared to Kv4.2^−/−^ mice (Fig. 9b). The NR1 subunit was used for the unambiguous identification of glutamatergic synapses containing NMDA receptors (Fig. 9a, b), and sampling of such synapses was over the entire dendritic tree of ITC neurons from proximal to distal sites. Our analysis revealed a significant increase in the frequency of NR1 immunoreactive synapses containing the NR2B subunit in Kv4.2^−/−^ mice compared to wild-type animals (Kv4.2^−/−^, 37.5 ± 5.0 %; Kv4.2^+/+^, 20.0 ± 2.7 %; *n* = 120 synapses per group, *p* = 0.026, Chi-square). On the other hand, the overall density of NR2B immunogold particles within postsynaptic specializations of NR2B-immunoreactive synapses in Kv4.2^−/−^ mice compared to wild-type animals was not significantly different (Kv4.2^−/−^, 4.20 ± 2.49 particles per 0.1 μm^2^, Kv4.2^+/+^ 3.94 ± 2.43 particles per 0.1 μm^2^; *n* = 30 synapses per group; *p* = 0.76, Mann–Whitney *U*). Likewise, the density of immunogold particles for NR1 did not differ in NR2B-immunoreactive synapses between Kv4.2^−/−^ mice and wild-type animals (Kv4.2^−/−^ 6.56 ± 4.83 particles per 0.1 μm^2^, Kv4.2^+/+^ 5.61 ± 4.60 particles per 0.1 μm^2^; *n* = 30 synapses per group; *p* = 0.25, Mann–Whitney *U*). These observations suggest that the number of synapses containing NR2B increases in Kv4.2^−/−^ mice, whereas the density of NR2B subunits likely remains unchanged.

## Discussion

This study highlights a number of novel aspects regarding the subcellular distribution of Kv4.2 pore-forming subunits and possible functional implications. We show here that Kv4.2 expression is restricted to somato-dendritic domains of ITC neurons and is differentially distributed in the extrasynaptic plasma membrane. Nearest neighbor analysis revealed a random distribution in the soma and a more homogeneous distribution in the dendrites. Although localized to extrasynaptic sites, Kv4.2 expression influenced the intrasynaptic expression of NMDA receptor subunits as the frequency of NR1 synapses containing the NR2B subunit was found increased in Kv4.2^−/−^ mice. Comparing the distribution profile of Kv4.2 subunits in ITC neurons to that of other central principal neurons revealed a highly cell-type and domain-specific organization in subunit distribution.

### ITC neurons in the rodent amygdala selectively express high levels of extrasynaptic Kv4.2 subunits

Our study revealed intense immunoreactivity for Kv4.2 subunits in all ITC subdivisions of the rat and mouse amygdala that was stronger than in any other amygdaloid area. This is in line with a previous report showing dense immunolabeling for Kv4.2 subunits in pericapsular ITC clusters of the rat amygdala (Dabrowska and Rainnie 2010). As these clusters lack Kv4.1 and Kv4.3 subunits, it is proposed that ITC neurons are equipped solely with homomeric Kv4.2 among *shal*-type channels.

In terms of subcellular distribution, we demonstrated that Kv4.2 is restricted to somato-dendritic domains of ITC neurons. Axonal compartments of this cell type lack Kv4.2 subunit expression. This indicates an important role for Kv4.2 channels in the processing and integration of glutamatergic inputs to ITC neurons. Our study also showed that dendrites have higher Kv4.2 subunit densities compared to the soma, and that these subunits do not appear to be associated with synaptic specializations. So far, data on the subcellular organization and synaptic localization of Kv4.2 subunits have been quite controversial with different patterns being described. With respect to overall subunit distribution, homogeneous and different non-homogeneous patterns were reported for distinct cell types. Clustering of Kv4.2 subunits was found, e.g., in supraoptic (Alonso and Widmer 1997), parasubicular (Jinno et al. 2005), cortical (Burkhalter et al. 2006) or olfactory neurons (Kollo et al. 2008). Our data showed that distinct patterns of Kv4.2 subunit organization indeed exist in different cell types and even in subdomains within a cell. Apart from the tight clustering of immunoparticles with NNDs below 40 nm, which was detected in all preparations, we found homogeneously distributed Kv4.2 subunits in the dendrites of ITC neurons and randomly distributed subunits in the dendrites of CA1 pyramidal neurons. On the other hand, we detected a random distribution of Kv4.2 subunits in the soma of ITC neurons and formation of loose clusters in the soma of CA1 pyramidal neurons and cerebellar granule cells. Since neuronal somata are often assumed to be equipotential with respect to electrophysiological properties, the net effect of such domain-specific distributions remains to be clarified.

With respect to synaptic localization, we consistently observed expression of Kv4.2 subunits in the extrasynaptic plasma membrane in all cell types analyzed. An early report by Alonso and Widmer (1997) suggested clustering of Kv4.2 subunits at postsynaptic sites in association with excitatory axon terminals and Shibasaki et al. (2004) showed activity-dependent targeting of Kv4.2 subunits to excitatory mossy fiber synapses in an in vitro cerebellar culture model. Kv4.2 subunits were also reported in inhibitory GABAergic synapses in different brain regions using pre-embedding approaches (Burkhalter et al. 2006; Jinno et al. 2005; Strassle et al. 2005). Our work did not show a synaptic localization of Kv4.2 subunits in either glutamatergic or GABAergic synapses of ITC neurons in agreement with a high-resolution study by Kerti et al. (2011) on Kv4.2 subunits in CA1 neurons. While a cell-type or circuit-specific association of Kv4.2 subunits with synapses cannot be ruled out, we believe that these subunits are predominantly extrasynaptic in central neurons as substantiated by the different ultrastructural immunolocalization techniques used in our study and by Nusser and coworkers (Kerti et al. 2011; Kollo et al. 2006, 2008). Inconsistencies might have resulted from the different methodological approaches applied. Kv4.2 channels in the extrasynaptic plasma membrane may function to counteract excitatory postsynaptic depolarizations and limit back-propagation of action potentials into distal dendrites, thereby reducing the excitability of the neuron and gating neuronal firing (Goldberg et al. 2003; Migliore et al. 1999). However, by controlling the temporal window at which back-propagating action potentials are boosted when paired with an excitatory postsynaptic potential (Magee and Johnston 1997), they may also contribute to synaptic plasticity.

Taken together, our data indicate a complex, cell type-specific distribution of Kv4.2 in central neurons and a lack of common organizational principles as it was reported for other types of potassium channels (Kaufmann et al. 2010; Luján 2010). The relatively even distribution of Kv4.2 subunits in the dendritic compartments of the ITC neurons further implies that they may display a global and input-independent impact on somato-dendritic signal integration and processing in this cell type.

### Labeling at the protoplasmic face of the membrane

It is noteworthy that the labeling for both, the Kv4.2_(209–225)_ and Kv4.2_(454–469)_, antibodies was observed at the protoplasmic (P-) face of the plasma membrane. While this was expected for the Kv4.2_(454–469)_ antibody, since it was directed against amino acids 454–469 located at the intracellular C-terminal domain of the protein, labeling with the Kv4.2_(209–225)_ antibody should have been visualized at the plasma membrane E-face as the antigenic peptide is in the extracellular S1–S2 loop (Birnbaum et al. 2004). The P-face labeling evident with the Kv4.2_(209–225)_ antibody was clearly not a staining artifact since the labeling pattern was identical to the one obtained with the Kv4.2_(454–469)_ antibody. Moreover, no labeling was observed in tissue samples from Kv4.2^−/−^ mice. Possible reasons for this unexpected finding are: i) a modification in the 3D structure of the channel subunit during a preparation step relocating the S1–S2 domain to the intracellular face of the membrane; or ii) the predicted transmembrane topology of the channel subunit does not correspond to its real conformation in vivo. Our findings warrant further studies to determine the exact conformation of the Kv4.2 protein in the plasma membranes of neurons.

### Modulation of NMDA synapses

Previous studies show that altering functional Kv4.2 expression levels and thus the *I*_*A*_ leads to altered NMDA receptor-dependent Ca^2+^ signaling and remodeling of NMDA synapses (Chen et al. 2006; Fontán-Lozano et al. 2011; Jung et al. 2008). Particularly, hippocampal pyramidal neurons exhibiting enhanced *I*_*A*_ showed a decrease in relative synaptic NR2B/NR2A subunit composition and did not exhibit LTP, whereas neurons with reduced *I*_*A*_ through genomic knockout of Kv4.2 led to an increased amount of synaptic NR2B and enhanced LTP (Jung et al. 2008). When using immunoblot analysis for studying NMDA synapse composition, total and synaptic levels of NR2B subunits were found increased in samples from Kv4.2^−/−^ mice, while levels of NR2A subunits remained unchanged (Jung et al. 2008). Noteworthy, not only genetic elimination of Kv4.2 but also pharmacological blockade of the Kv4.2-mediated *I*_*A*_ facilitated the induction of LTP at excitatory synapses and increased the induction threshold for LTD (Chen et al. 2006; Zhao et al. 2011). Although it was speculated that Kv4.2 controlled synaptic NMDA receptor expression and plasticity as an integral part of a synaptic complex, we localized Kv4.2 subunits to the extrasynaptic plasma membrane in ITC neurons. Still, we found a significant increase in NMDA synapses containing the NR2B subunit in Kv4.2^−/−^ mice compared to wild-type animals. This indicates an indirect, yet strong impact of Kv4.2 on the NMDA receptor subunit composition and probably on synaptic plasticity in ITC neurons. Ca^2+^-dependent potentiation of NMDA synapses potentially relies on the NR2B subunit content as NR2B-mediated currents have slower kinetics than NR2A-mediated currents, allowing for greater temporal summation and Ca^2+^ influx (Malenka and Nicoll 1999). In fact, ifenprodil, a use-dependent NR2B-selective blocker (Williams 1993), prevented LTP induction in young CA1 neurons in an organotypic slice culture model (Barria and Malinow 2005). Although several studies have dealt with the role of *I*_*A*_ in controlling neuronal excitability and synaptic plasticity in central neurons, the actual impact of *I*_*A*_ on synaptic plasticity in ITC neurons can only be surmised. In the hippocampus and neocortex, *I*_*A*_ is synaptically regulated and controls the back-propagation of action potentials into dendrites of pyramidal cells and interneurons, which activate Ca^2+^, Na^+^ and NMDA receptor channels and influence the dendritic integration of synaptic inputs (Cai et al., 2004; Goldberg et al. 2003; Hoffman et al. 1997; Losonczy and Magee 2006). Our data show that Kv4.2 is highly expressed in ITC dendrites where the expressed Kv4.2 currents may have similar properties, and thus contribute to synaptic plasticity. Since ITC neurons are required for fear learning and extinction of fear (Busti et al. 2011; Likhtik et al. 2008), further experiments addressing the role of Kv4.2 in Pavlovian fear conditioning are warranted.

## Conclusion

We found that the Kv4.2 pore-forming A-channel subunit is densely expressed in ITC neurons and localized to the extrasynaptic plasma membrane of ITC somata and dendrites. Kv4.2 gene deletion induces an increase in the frequency of NMDA synapses containing the NR2B subunit, which indicates a strong impact of Kv4.2 expression on synaptic integration at this site, central to signal transduction and processing from the basolateral amygdala to the central nucleus.

## Abbreviations

I_A_: A-type K^+^ current
ITC: Intercalated cell cluster
K_V_4.2: Voltage-gated potassium channel type-4.2
LTD: Long-term depression
LTP: Long-term potentiation
MOR: μ-Opioid receptor
NND: Nearest neighbor distance
NR1: NMDA-type glutamate receptor subunit 1
NR2B: NMDA-type glutamate receptor subunit 2B
PSD-95: Postsynaptic density protein 95
SDS-FRL: Sodium lauryl sulfate-digested freeze-fracture replica labeling
